# Manic episode in patient with bipolar disorder and recent multiple sclerosis diagnosis: A case report

**DOI:** 10.1097/MD.0000000000022823

**Published:** 2020-10-16

**Authors:** Simon Yang, Lora Wichser

**Affiliations:** a University of Minnesota Medical School; b Department of Psychiatry, University of Minnesota, Minneapolis, MN.

**Keywords:** bipolar disease, mania, multiple sclerosis, neuroimaging, mood disorder

## Abstract

**Introduction/Rationale:**

Multiple sclerosis (MS) is associated with a higher prevalence of mood and psychiatric disorders, such as bipolar disorder (BD). While mania is most often associated with BD, MS can also induce manic symptoms. However, it is crucial to distinguish which condition is causing mania since medical management is different based on its etiology. Herein, we report a case of a manic episode in a middle-aged female with a prolonged history of BD who received a recent diagnosis of MS 1 year ago.

**Patient Concerns:**

A 56-year-old female presented with an episode of mania and psychosis while receiving a phenobarbital taper for chronic lorazepam use. She had a prolonged history of bipolar type 1 disorder and depression. She showed optic neuritis and was diagnosed with MS a year prior.

**Diagnoses:**

The patient was diagnosed with BD-induced mania based on the absence of increased demyelination compared to previous MRI and lack of new focal or lateralizing neurologic findings of MS.

**Interventions:**

Lithium was given for mood stabilization and decreased dosage of prior antidepressant medication. Risperidone was given for ongoing delusions.

**Outcomes:**

After 8 days of hospitalization, patient's mania improved but demonstrated atypical features and ongoing delusions. She was discharged at her request to continue treatment in an outpatient setting.

**Conclusion/Lesson:**

In BD patients with an episode of mania, MS should be included in the differential, since both conditions can cause manic symptoms. The origin of mania should be delineated through a detailed neurological exam, neuroimaging, and thorough patient-family psychiatric history for appropriate clinical treatment.

## 1 Introduction

Multiple sclerosis (MS) is an inflammatory autoimmune disease that focally damages the white matter in the brain and spinal cord.^[1]^ It affects 1 in 1000 people and is the most common central nervous system disease for young adults in the Western world.^[2]^ Initially, neurological symptoms are transient due to remyelination, but repeated demyelination progressively leads to diffuse and chronic neurodegeneration. Furthermore, previous studies have shown increased psychiatric symptoms and higher prevalence of psychiatric and mood disorders.^[3]^

Bipolar disorder (BD) is a mood disorder characterized by extreme mood fluctuations with episodes of mania or hypomania and depression. Mania, a hallmark of BD, is when the patient is in a state of elevated mood and energy, during which the patient reports symptoms such as euphoria or irritable mood, racing thoughts, overactivity, and reduced need for sleep. BD affects more than 1 in 100 people worldwide.^[4]^

The prevalence of BD in MS patients has been reported to be twice than that of the general population.^[5]^ For patients diagnosed with BD and MS, there is no clear method to distinguish whether mania was induced from BD or from a MS flare-up. However, it is important to discern the cause of manic episode since management is different for BD-induced mania vs MS-induced mania. Herein, we describe a patient diagnosed with BD that later developed MS who presented to us during a manic episode. Through this case, we aim to examine the BD versus MS origins of manic episodes and discuss relevant literature.

## 2 Case Presentation

The patient was a 56-year-old female who came to us during an episode of mania and psychosis while receiving treatment at an addiction treatment center where she was taking a phenobarbital taper for chronic lorazepam use. She displayed symptoms of aggressive posturing, verbal abuse to staff, delayed response, and racing thoughts. She did not describe suicidal thoughts. She had 4 prior psychiatric hospitalizations. At age 33, she exhibited depression, anxiety, and paranoia that lead to her first hospitalization. At age 44, she attempted suicide via acetaminophen overdose. Her first reported manic episode was at age 45, during which bipolar type 1 disorder was considered as her differential and subsequently diagnosed. Her symptoms accompanied delusions during this episode, without suicidal ideation. Her most recent hospitalization was at age 49 for depression and paranoia with delusions of being wiretapped and people reading her mind. At age 55, the patient presented with optic neuritis and diagnosed with MS after a lumbar puncture showed oligoclonal bands.

Family history revealed depression in father and alcohol use disorder in mother. Past medical history described an acute onset dizziness when moving eyes left to right or vice versa and when standing up from a lying position.

Neurology consult found no focal or lateralizing findings. MRI analysis showed greater than 15 foci of T2 hyperintensity within white matter where some lesions were within periventricular and juxtacortical white matter of both cerebral hemispheres, consistent with a demyelinating disease. A single focus of enhancement in the posterior corona radiata was suggestive of active demyelination. No demyelinating signs were seen in the thoracic spine. However, no significant difference was seen compared to previous MRI.

During the present hospitalization, patient's prior bupropion was reduced due to concern for further mania activation. Lithium 600 mg twice a day was prescribed for mood stabilization. Risperidone 0.5 mg at bedtime was prescribed for ongoing delusions. Patient was not taking scheduled steroids prior to admission. After 8 days of hospitalization, patient's mania improved but demonstrated atypical features, such as absence of pressured speech, grandiosity, risk taking or sleep pattern changes. Per a family member's report, patient stated that she was in a movie and that everyone else was acting around her. Patient requested discharge to continue treatment in an outpatient setting.

## 3 Discussion

Although neurological symptoms of MS have been extensively studied, the psychiatric effects of MS are relatively less elucidated, despite the fact that the association of MS and psychiatric symptoms observed as early as 1872 by Jean-Martin Charcot.^[6]^ In 1986, Schiffer et al suggested an association between BD and MS after identifying 10 patients with both BD and MS, out of more than 700,000 individuals, when epidemiologic data expected to find only 5.4 patients.^[7]^ Co-occurrences of BD and MS have been reported infrequently through case studies. Recently, Carta et al conducted a case control study with 201 MS patients that examined the risk of BD in MS patients and reported OR of 44.4 for bipolar spectrum disorders. Specifically, bipolar type 2 diagnoses (7.5%) was more frequent than bipolar type 1 diagnoses (0.99%).^[8]^

The exact underlying mechanism and pathophysiology of BD and MS co-presentation is yet to be established. It is unknown whether BD is an early manifestation of MS or if both diseases share a common underlying cause presenting at similar timelines. More recent studies have shown genetic associations between BD and MS in human leukocyte antigen (HLA) DR2 gene and mitochondrial transcriptomes.^[9,10]^ Further understanding of the etiology of this association may elucidate whether there are synergistic effects or crosstalk between MS and BD therapeutics.

While mania is a hallmark symptom of BD, MS can also exhibit a range of psychiatric symptoms including mania, euphoria, depression, hallucinations, and episodes of pathologic laughing and weeping, which is coined as ‘pseudobulbar effects.’^[11]^ Focal neuronal demyelination in MS patients may interfere with communication between frontal lobe brain regions responsible for emotion and manifests as emotional lability and exaggerated emotions, common symptoms in a manic or depressive episode.^[12]^ Features of MS flare-up mania are no different than those of non-MS mania. However, the incidence of psychosis has been reported to be less common in MS.^[13]^

Differentiating the cause of the manic episode is of clinical significance as the treatment plan differs between a MS flare-up and a BD manic episode. For instance, while lithium and sodium valproate have been shown to be effective in treating mania in BD, no controlled trials of its efficacy in mania in MS patient has been published.^[14]^ Additionally, manic episodes due to medications cannot be precluded. Steroid treatment in MS patient may often cause a moderate degree of mania.^[15]^ Patients with a family history of alcohol use disorder or other affective disorder are more vulnerable to this cause.^[15]^ Other medications, such as tizanidine, baclofen, and dantrolene, can also cause hypomania following their use.^[16]^ Manic symptoms due to medications are often dose-dependent and manifest soon after initiating the medication.^[16]^

Detailed neurologic tests or neuroimaging can often help differentiate the cause of a manic episode. MS flare-ups often manifest with increased focal neurological symptoms including visual loss, fatigue, urinary incontinence, and cognitive impairment, in addition to any of the afore-mentioned mood symptoms. Additionally, MS flare-ups may show an increased degree of demyelination on MRI compared to prior images.

Both MS and BD-onset mania have been reported to show white matter changes on MRI by Young et al.^[17,18]^ Especially, MS patients with mania and psychotic symptoms were shown to have plaques located in the bilateral temporal horn areas.^[14]^ Neuroimaging of BD patients without MS has been more complex. Several studies proposed increased white matter and periventricular hyperintensities in these patients.^[19,20]^ McDonald et al reported increased subcortical hyperintensities in T2 weighted MRI in late-onset BD patients.^[19,21]^ Dupont et al reported increased white matter hyperintensities in early-onset BD patients.^[19,22]^ Altshuler et al reported no significant difference white matter hyperintensities, but increased periventricular hyperintensity in BD type 1 patients.^[19,23]^

In our case, the absence of aforementioned focal or lateralizing finding in MS during the neurological exam, absence of increased demyelination compared to previous MRI, and family history of psychiatric disorders decreased the likelihood of her current symptoms representing a MS flare-up and was more consistent with BD-induced mania. Additionally, patient was not taking mania-inducing medications such as steroids, tizanidine, baclofen, or dantrolene. Patient's symptoms improved with lithium treatment. The patient's MRI showed increased white matter and periventricular T2 hyperintensity. However, no plaques at bilateral temporal horn areas were identified. Considering that her symptom onset was during a phenobarbital taper for chronic benzodiazepine use, her mania may have been a BD manic episode triggered by her benzodiazepine withdrawal directly or exacerbated from withdrawal symptoms, such as poor sleep and increased anxiety.

The ages at which this patient's illnesses presented - BD type I onset at age 45 preceding MS onset at age 55, is of particular note in relation to previous case reports. Marangoni et al identified case reports of 26 patients who had BD onset clearly preceding MS, via a PubMed search from inception to 2014.^[24]^ The study showed an average of 5 years difference between BD and MS onset. The majority of these patients were found to have BD type I, where 25 patients had BD type I and 1 patient had BD type II with rapid cycling. Three cases reported family history of MS and 6 cases reported psychiatric family history. The study also noticed increased white matter lesions in periventricular and subcortical white matter – which was consistent with our case - as well as in the centrum semiovale, frontal, parietal, and temporal lobes. However, it did not identify association between certain BD type to MS types nor association between certain BD types with patterns of white matter lesions.

While the study had insufficient data to formulate a valid hypothesis, the study found that BD-preceded-MS had a higher age of both BD and MS onset compared to the age of onset of the combined pool of patients with BD and MS regardless of onset order. The study also suggested that later onset of MS may be associated with co-occurrence with BD. This case report, where the patient was diagnosed with BD and MS relatively later than the common age of onset of 20s or 30s, substantiates these trends found in previous case reports by Marangoni et al and speculates that late onset of BD or MS may be associated with BD-MS comorbidity. Past reports showed cases where acute psychotic symptoms led to MS diagnosis, which were coined as “inaugural manifestations” to MS.^[25]^ Future research into the timing of onset can elucidate whether late diagnosis of mood or psychotic disorders can be early signs of comorbidity with MS.

## 4 Conclusion

In patients with co-occurrence of BD and MS, there is currently no clear guideline to discern the origin of manic episodes. However, it is important to attempt to discern the predominant cause of the manic episode through detailed patient history, neurologic exam, and neuroimaging, as it can affect treatment plans. Additionally, the presented case, along with previous cases of BD-preceding-MS correlating with generally later age of onset of BD and MS, may be a future direction for further investigation.

## Author contributions

**Conceptualization:** Simon Yang.

**Supervision:** Lora Wichser.

**Writing – original draft:** Simon Yang.

**Writing – review & editing:** Simon Yang, Lora Wichser.
